# Acute and probable chronic Q fever during anti-TNFα and anti B-cell immunotherapy: a case report

**DOI:** 10.1186/1471-2334-14-330

**Published:** 2014-06-15

**Authors:** Teske Schoffelen, Alfons A den Broeder, Marrigje Nabuurs-Franssen, Marcel van Deuren, Tom Sprong

**Affiliations:** 1 Department of Internal Medicine, Radboud university medical center, Nijmegen, The Netherlands; 2 Department of Rheumatology, Sint Maartenskliniek, Nijmegen, The Netherlands; 3 Department of Medical Microbiology and Infectious Diseases, Canisius Wilhelmina Hospital, Nijmegen, The Netherlands; 4 Department of Internal Medicine, Canisius Wilhelmina Hospital, Nijmegen, The Netherlands

**Keywords:** Q fever, *Coxiella burnetii*, Immunosuppression, Rheumatoid arthritis, Tumor necrosis factor-α, Anti-TNFα, Etanercept, Adalimumab, Rituximab, Serology

## Abstract

**Background:**

Q fever is caused by the intracellular bacterium *Coxiella burnetii*. Initial infection can present as acute Q fever, while a minority of infected individuals develops chronic Q fever endocarditis or vascular infection months to years after initial infection. Serology is an important diagnostic tool for both acute and chronic Q fever. However, since immunosuppressive drugs may hamper the humoral immune response, diagnosis of Q fever might be blurred when these drugs are used.

**Case presentation:**

A 71-year-old Caucasian male was diagnosed with symptomatic acute Q fever (based on positive *C. burnetii* PCR followed by seroconversion) while using anti-tumor necrosis factor-α (anti-TNFα) drugs for rheumatoid arthritis (RA). He was treated for two weeks with moxifloxacin. After 24 months of follow-up, the diagnosis of probable chronic Q fever was established based on increasing anti-*C. burnetii* phase I IgG antibody titres in a immunocompromised patient combined with clinical suspicion of endocarditis. At the time of chronic Q fever diagnosis, he had been treated with anti B-cell therapy for 16 months. Antibiotic therapy consisting of 1.5 years doxycycline and hydroxychloroquine was started and successfully completed and no signs of relapse were seen after more than one year of follow-up.

**Conclusion:**

The use of anti-TNFα agents for RA in the acute phase of Q fever did not hamper the *C. burnetii*-specific serological response as measured by immunofluorescence assay. However, in the presented case, an intact humoral response did not prevent progression to probable chronic *C. burnetii* infection, most likely because essential cellular immune responses were suppressed during the acute phase of the infection. Despite the start of anti-B-cell therapy with rituximab after the acute Q fever episode, an increase in anti-*C. burnetii* phase I IgG antibodies was observed, supporting the notion that *C. burnetii* specific CD20-negative memory B-cells are responsible for this rise in antibody titres.

## Background

Q fever is caused by the intracellular growing bacterium *Coxiella burnetii*[1]*.* Acute Q fever is a (self-limiting) febrile illness, but can present as pneumonia or hepatitis. Chronic Q fever presents most often as an endovascular infection, i.e. endocarditis or mycotic aneurysm or infected vascular graft, which has a high mortality if left untreated [2,3]. Risk factors are underlying valvular defects, or pre-existing vascular aneurysm or prosthesis.

Immunosuppression is another stated risk factor for chronic Q fever, as some immunosuppressive drugs decrease protective cellular responses against intracellular growing bacteria. This risk factor has thus far been poorly documented, but recently we confirmed that patients with rheumatoid arthritis (RA) using immunosuppressive drugs are indeed at increased risk of developing chronic Q fever [4].

Clinical signs of *C. burnetii* infection are often nonspecific, and the diagnosis of acute or chronic Q fever is heavily based on measurement of antibody titres [5,6], complemented by the direct detection of the micro-organism by polymerase chain reaction (PCR) [7,8]. Serologic criteria for Q fever consist of measurement of antibodies against the two antigenic forms of *C. burnetii,* phase I and II organisms, with high anti-*C. burnetii* phase I IgG titres - in the absence of acute Q fever – pointing to a chronic infection. The appropriate cut-off titre that differentiates it from a past cleared infection is debated; currently proposed cut-offs are 1:1,024 or 1:1,600 [6,9].

The diagnosis of Q fever in hosts on immunosuppressive drugs may be complicated, because these drugs can inhibit antibody responses and therefore hamper correct diagnosis based on serologic results. Also the immune-mediated disease itself, for which these drugs are prescribed, may contribute to inadequate immune responses to infection [10,11].

Here we present a case history of a patient with RA who had an episode of acute Q fever while being treated with anti-tumor necrosis factor-α (anti-TNFα) medication, and who developed probable chronic Q fever over the subsequent two years while using the anti-B-cell monoclonal antibody rituximab. The case highlights the importance of cellular and humoral immune response modifying agents in the natural course of *C. burnetii* infections and the possible pitfalls of the use of serological methods to detect the stage of disease.

## Case presentation

### Acute Q fever

In May 2009, during the Dutch Q fever epidemic, a 71-years-old rheumatoid factor and anti-CCP positive RA patient living in the Q fever high incidence area, presented with 8 days of fever and a non-productive cough. He was receiving anti-rheumatic treatment including etanercept (an anti-tumor necrosis factor-α [anti-TNFα] agent) and prednisone. He had a history of atrial fibrillation, but no underlying valvulopathy. Physical examination and a chest X-ray were compatible with a pulmonary infiltrate. No murmurs were heard upon cardiac auscultation. Laboratory investigations revealed increased C-reactive protein (CRP, 285 mg/L), a normal leukocyte count (5.2×10^9^/L) and normal values for renal function and liver enzymes. PCR (real-time PCR targeting the IS1111a insertion element [12]) for *C. burnetii* on plasma turned out to be positive. However, serology (immunofluorescence assay [IFA, Focus Diagnostics, Cypress, USA]) was negative for IgM as well as for IgG against phase I and II *Coxiella burnetii*. The diagnosis of acute Q fever was made and treatment with moxifloxacin 400 mg daily for 14 days was started. Two weeks later, seroconversion was observed with anti-phase I and II IgM titres of 1:4096 and 1:16384 respectively.

### Diagnosis and treatment of probable chronic Q fever

After quick recovery, the patient was followed-up to monitor for possible progression to chronic Q fever. During this period, the anti-rheumatic treatment had been switched by the rheumatologist from etanercept to adalimumab (another anti-TNFα agent), and subsequently – 8 months after the acute Q fever episode – to rituximab. The latter, an anti-CD20 anti B-cell monoclonal antibody, had resulted in adequate suppression of the rheumatic activity.

**Figure 1:**
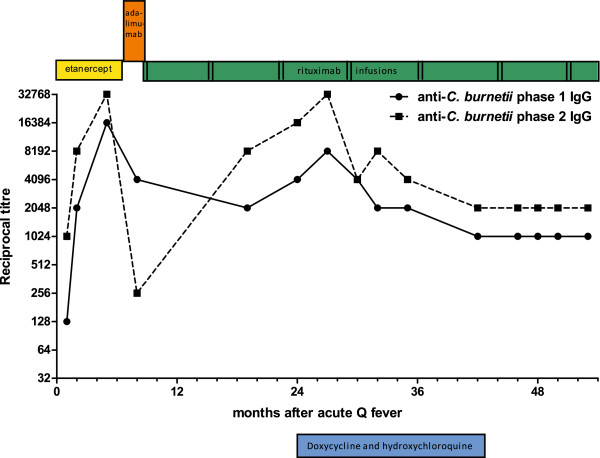
**Q fever serology during follow-up after acute Q fever in a patient with anti-rheumatic drugs.** Serological titres of anti-*Coxiella burnetii* phase I and phase II IgG (as measured by immunofluorescence assay) during follow-up after acute Q fever (t = 0) of a patient using subsequently etanercept, adalimumab and rituximab as biological disease-modifying anti-rheumatic drugs. Rituximab was given with 7 months intervals, each time two dosages with two weeks interval. At t = 24 the diagnosis probable chronic Q fever was established followed by anti-microbial treatment with doxycycline and hydroxychloroquine for 1.5 years.

As can be seen in Figure 1, anti-phase I IgG antibodies titres had not decreased below 1:1024 after more than one year, and continued to increase to 1:4096 at 24 months, suggesting development of chronic Q fever. At that moment, the patient had complaints of general fatigue but no fever, night-sweats or cardiac problems. On physical examination, however, a grade 2/6 aortic systolic murmur was audible. Transesophageal echocardiography (TEE) showed no signs of endocarditis, but an echogenic mitral annulus and a trace of mitral valve insufficiency. Laboratory investigation showed an ESR 40 mm/hr with CRP < 2 mg/L. PCR for *C. burnetii* in plasma was repeatedly negative. Abdominal ultrasound did not reveal an aortic aneurysm. Positron-emission tomography (PET)-scanning showed hilar lymphadenopathy but no other abnormalties. Endobronchial biopsy of the hilar lymph nodes was PCR negative for *C. burnetii* and showed no signs of malignancy. Because of the increasing anti-phase I IgG titres in this immunocompromised patient, in combination with nonspecific complaints and the new cardiac murmur , the diagnosis of ‘probable chronic Q fever’ was made [13] and the patient was started on doxycycline 200 mg daily combined with hydroxychloroquine 200 mg three times daily for 1.5 years. Intermittent courses with rituximab were continued as anti-rheumatic treatment, in combination with azathioprine. During antibiotic treatment, the anti-phase I IgG titres decreased from 1:8192 to 1:1024. The patient experienced improvement from his fatigue. After 1.5 years treatment, PET-scanning and TEE were unchanged. After discontinuation of antibiotics, the serological and clinical follow-up was pursued and is still continuing, with no relapse after more than one year.

## Discussion

We report on an immunocompromised patient, followed-up from the start of a symptomatic acute Q fever episode to the development and treatment of probable chronic Q fever. Our case illustrates three interesting aspects of diagnosis and treatment of Q fever in an immunocompromised host. First, we noticed that, despite the presence of RA and use of anti-TNFα agents, the humoral response to the initial *C. burnetii* infection was not impaired. Secondly, in spite of adequate treatment, the acute Q fever progressed to probable chronic Q fever endocarditis, which suggests incomplete clearance of the infection during the acute stage. Finally, the increase in phase I IgG titres, which serves as a marker for chronic Q fever, occurred under treatment with anti B-cell immunotherapy which was started after the acute Q fever episode.

In the presented case, a definite diagnosis of chronic Q fever could not be made. *C. burnetii* DNA was not detected in blood on several occasions and echocardiography did not show major signs of endocarditis. Nevertheless, chronic Q fever was clinically highly suspected. The patient was followed-up after acute Q fever, and we observed that the antibody titres were falling after they had peaked following the acute Q fever episode, only to rise again after 18 months. This increase in antibody titres was accompanied by aspecific complaints of fatigue and a newly diagnosed cardiac murmur. According to the Dutch guidelines, this was a diagnosis of probable chronic Q fever [13], and the patient received antibiotic treatment as considered appropriate.

Interestingly, we observed a normal antibody response in the acute phase of the Q fever infection under anti-TNFα therapy. This is in line with previous studies which have shown that anti-TNFα therapy does not prevent serologic responses to influenza vaccination [14,15], although titres may be somewhat lower. Clearly, in our case, this normal antibody response did not prevent the development of persistent *C. burnetii* infection, as complete clearance might depend more on cellular immune responses.

Indeed, to constrain intracellular *C. burnetii* infections, a cellular immune response is crucial and TNFα is a key cytokine in this response. In-vitro studies showed that TNFα mediates interferon-gamma induced intracellular killing of *C. burnetii* in monocytes through apoptosis [16]. TNF knock-out mice infected with *C. burnetii* develop early bactaeremia and severe heart lesions [17]. Infection risk due to decreased cellular immunity in anti-TNFα treated patients, has been shown for other intracellular infections, most notably *Mycobacterium tuberculosis*, but also *Listeria monocytogenes* and *Salmonella enterica*, and for herpesviridae [18-21].

Rituximab, an anti-CD20 monoclonal antibody, is used for the treatment of RA patients failing on TNFα blockers. Rituximab depletes circulating CD20-positive B-cells for a period of six to nine months [22]. As a consequence, patients on rituximab therapy have an impaired antibody response to neo-antigens. Existing plasma cells and memory B-cells, which do not express CD20, are not affected by rituximab [23]. During the development from acute to chronic Q fever, there is an ongoing infection with presumably increasing concentrations of *C. burnetii* antigens. Because plasma cells do not express B-cell receptors at their surface, which makes them incapable to respond to alterations in antigen concentrations, we assume that the rise of anti-*C. burnetii* IgG titres in our patient originated from stimulation of memory IgG B-cells by increased concentrations of recall antigens. This intact response to recall antigens after rituximab has been observed for patients receiving vaccinations [24], but has never been documented after natural infection.

Our results indicate that anti-*C. burnetii* phase I IgG antibody titres can be used as a marker for progression to chronic Q fever and the subsequent response to therapy in patients in whom B-cell depleting therapy is started after initial exposure. However, it is likely that B-cell depleting medication during first contact with neo-antigens of *C. burnetii* will seriously hamper the development of an antibody response and the diagnosis of Q fever based on serological titres.

## Conclusions

The use of anti-TNFα agents for RA in the acute phase of Q fever does not seem to impede the *C. burnetii*-specific serological response. However, in the presented case, an intact humoral response did not prevent progression to probable chronic *C. burnetii* infection, most likely because essential cellular immune responses were suppressed in the acute phase of the infection. Even though anti-B-cell therapy with rituximab was started after the acute Q fever episode, an increase in anti-*C. burnetii* phase I antibodies was observed, supporting the notion that *C. burnetii* specific CD20-negative memory B-cells are responsible for this rise in antibody titres.

### Consent

Written informed consent was obtained from the patient for publication of this Case report. A copy of the written consent is available for review by the Editor of this journal.

## Competing interests

All authors declare that they have no competing interests.

## Authors’ contributions

TSc collected the data on this patient and drafted the manuscript. TSp collected the data on this patient and helped to draft the manuscript. MvD helped to draft the manuscript. All authors interpreted the data. All authors read, edited and approved the final manuscript.

## Pre-publication history

The pre-publication history for this paper can be accessed here:

http://www.biomedcentral.com/1471-2334/14/330/prepub
